# PRMT1 suppresses ATF4-mediated endoplasmic reticulum response in cardiomyocytes

**DOI:** 10.1038/s41419-019-2147-3

**Published:** 2019-12-02

**Authors:** Myong-Ho Jeong, Hyeon-Ju Jeong, Byeong-Yun Ahn, Jung-Hoon Pyun, Ilmin Kwon, Hana Cho, Jong-Sun Kang

**Affiliations:** 1 Department of Molecular Cell Biology, Sungkyunkwan University, Suwon, Republic of Korea; 2 Single Cell Network Research Center, Sungkyunkwan University, Suwon, Republic of Korea; 3 Division of Cardiovascular Diseases, Center for Biomedical Sciences, National Institute of Health, Cheongju, Chungbuk Korea; 4 Department of Anatomy and Cell Biology, Sungkyunkwan University, Suwon, Republic of Korea; 5 Department of Physiology, Sungkyunkwan University, Suwon, Republic of Korea

**Keywords:** Cell death, Heart failure

## Abstract

Endoplasmic reticulum (ER) stress signaling plays a critical role in the control of cell survival or death. Persistent ER stress activates proapoptotic pathway involving the ATF4/CHOP axis. Although accumulating evidences support its important contribution to cardiovascular diseases, but its mechanism is not well characterized. Here, we demonstrate a critical role for PRMT1 in the control of ER stress in cardiomyocytes. The inhibition of PRMT1 augments tunicamycin (TN)-triggered ER stress response in cardiomyocytes while PRMT1 overexpression attenuates it. Consistently, PRMT1 null hearts show exacerbated ER stress and cell death in response to TN treatment. Interestingly, ATF4 depletion attenuates the ER stress response induced by PRMT1 inhibition. The methylation-deficient mutant of ATF4 with the switch of arginine 239 to lysine exacerbates ER stress accompanied by enhanced levels of proapoptotic cleaved Caspase3 and phosphorylated-γH2AX in response to TN. The mechanistic study shows that PRMT1 modulates the protein stability of ATF4 through methylation. Taken together, our data suggest that ATF4 methylation on arginine 239 by PRMT1 is a novel regulatory mechanism for protection of cardiomyocytes from ER stress-induced cell death.

## Introduction

The endoplasmic reticulum (ER) is a pivotal organelle accountable for cellular housekeeping functions, including protein folding/maturation, lipids/steroids biosynthesis, as well as calcium homeostasis^1^. Perturbation of ER homeostasis causes the accumulation of unfolded and misfolded proteins, referred to as ER stress, leading to activation of an unfolded protein response (UPR)^2–4^. The UPR pathway restores protein homeostasis via suppression of general protein translation, induction of ER-related molecular chaperones and ER-associated degradation, eventually leading to cell survival^5^. Accumulating evidence suggest that ER stress deregulation has been implicated in various heart diseases such as cardiac hypertrophy, ischemic heart disorders, and arrhythmias^6–9^. ATF4 is a master regulator for ER stress response. Under prolonged ER stress, ATF4 induces CHOP or NOXA thereby initiating a proapoptotic pathway^10,11^. Recently, it is reported that ATF4 and CHOP triggers the protein synthesis possibly causing proteotoxicity^12^. This was underlined by a study showing the resistance against the pressure overload-induced heart failure in mice lacking CHOP^13^. Furthermore, ATF4 overexpression promoted cardiomyocyte death^14^. Thus, understanding the regulatory mechanism of ATF4/CHOP pathway is important to develop the therapeutic strategy against cardiomyopathy. Extensive studies have revealed that the ATF4 stability and target selectivity is controlled by posttranslational modifications, including ubiquitination, acetylation, phosphorylation, and arginine methylation^15,16^.

Protein arginine methylation is one of the posttranslational modifications that regulate histone or nonhistone substrates in various cellular responses. PRMT1 is a major cellular arginine methyltransferase that controls wide range of tissue homeostasis^17–20^. Recently, two reports demonstrate the importance of PRMT1 in cardiac function^21,22^. Cardiac-specific PRMT1 ablation exhibits the contractile dysfunction around 5-weeks of age and die within 2-months with dilated cardiomyopathy and heart failure. Mechanistic studies have revealed that the aberrant regulation of alternative splicing transition and CaMKII activity underlying the contractile dysfunction of PRMT1-deficient heart^21,22^. Since PRMT1-deficient heart caused dilated cardiomyopathy accompanied by cardiomyocyte death, PRMT1 might be important for cardiomyocyte survival. Recent reports have proposed a role for PRMT1 in ER stress-mediated cell death. PRMT1 can methylate ATF4 in a tumor suppressor BTG1-dependent manner that induces cell death to prevent cellular malignancy^16^. Furthermore, PRMT1 deficiency attenuates palmitate-induced ER stress activation and mesangial cell apoptosis^23^. These evidences suggest that PRMT1 may act as an important regulator of ER stress controlling cell survival and death.

In this study, we focus on the role of PRMT1 in cardiac ER stress and cell death. PRMT1 inhibition by a PRMT1 specific inhibitor furamidine or shRNA in newborn rat ventricular myocyte (NRVM) and H9C2 cardiomyocytes enhances ATF4/CHOP pathway. In alignment with the in vitro results, the abnormal cell death is observed in 2-week-old PRMT1-deficient hearts. The global gene expression profile of 2-week-old control and PRMT1-deficient hearts shows that PRMT1 deletion evokes upregulation of ATF4/CHOP pathway resembling that of wildtype hearts treated with an ER stress inducer, tunicamycin (TN). Consistently, PRMT1-deficient hearts exhibited aggravated cell death in response to the TN treatment. The methylation of ATF4 at arginine residue 239 (R239) by PRMT1 appears to be critical for the control of ATF4 activity and stability. ATF4 mutant of R239 to lysine switch (R239K) exacerbated TN-triggered cell death. Taken together, these data suggest that PRMT1 suppresses ER stress response by ATF4 methylation in cardiomyocytes.

## Methods

### Animal studies

*PRMT1*^*f/f*^ mice were maintained as previously described^21,24^. To generate cardiomyocyte-specific PRMT1 null mice, *PRMT1*^*f/f*^ mice were crossed with *PRMT1*^*f/+*^ carried with Myh6-Cre gene (*PRMT1*^*f/+;Myh6-Cre;*^). Tg(Myh6-cre)2182Mds/J mice were obtained from Jackson laboratory. To investigate the role of PRMT1 in ER stress response, *PRMT1*^*f/f*^ (WT) and *PRMT1*^*f/f/Myh6-Cre*^ (cKO) mice were intraperitoneally injected with TN (1.5 mg/kg body weight) or control vehicle (150 mM of Dextrose) and 16 h later, hearts were harvested for the molecular analysis. This study was reviewed and carried out in accordance with the Institutional Animal Care and Use Committee of Sungkyunkwan University School of Medicine.

### Immunostaining

Immunostaining of cardiac tissue was performed as previously described^25^. To analyze the cell death in mouse heart samples, terminal deoxynucleotidyl transferase dUTP nick end labeling (TUNEL) assay was performed using Click-iT®TUNEL Alexa Fluor® kit according to manufacturer’s protocol (Invitrogen, C10246). Briefly, deparaffinized samples were incubated with TdT enzyme at 37 °C for 1 h and signals were developed with reaction buffer (Alexa Fluor 594) for 30 min in dark room. After wash the samples with 3% bovine serum albumin (BSA) solution, nucleus was stained with Hoechst 33342 and analyzed with confocal microscopy. For immunocytochemistry, cells were fixed with 4% PFA for 10 min and permeabilized with 0.1% Triton X-100 in phosphate-buffered saline (PBS) for 10 min. Cells were then blocked with blocking buffer (2% BSA in 0.1% PBST) for 30 min and incubated with primary antibodies diluted in blocking buffer for overnight at 4 °C. Images were analyzed with an LSM-710 confocal microscope system (Carl Zeiss), Tissue FAXS i8 plus (TissueGnostics) or Nikon ECLIPS TE-2000U and processed with ZEN software (Carl Zeiss), TissueQuest analyzer (TissueGnostics), NIS-Elements F (Nikon) or image J software.

### Cell culture, transfection, and luciferase assay

HEK293T and H9C2 rat embryonic cardiomyocytes were cultured as previously described^21^. Isolation and culture of NRVM was performed as previously described^26^. For transfection experiments, Lipofectamine2000 (Invitrogen, 11668) or Polyethylenimine (1 mg/mL, Sigma-Aldrich) were used. To deplete PRMT1, NRVMs were infected with adenovirus expressing control scrambled shRNA or PRMT1 shRNA as previously described^21^. For ATF4 knockdown, NRVM and H9C2 cells were transfected with control scrambled siRNA or siATF4 (CREB-2: sc-35113, Santa Cruz) using Lipofectamine RNAiMAX according to manufacturer’s instruction. For overexpression studies, the expression vectors for PRMT1, ATF4, or ATF4 mutants were transfected into HEK293T, H9C2, or NRVM cells using polyethylenimine or Lipofectamine 2000. To analyze the ER stress, NRVM or H9C2 cells were treated with vehicle DMSO or 2.5–10 μg/ml TN in combination with either vehicle DMSO, 50 μM DS-437 or 20 μM furamidine for 24–48 h. For the luciferase assay, H9C2 cells were transfected with the expression vectors for ERSE-driven luciferase (CCS-2032L, QIAGEN) or ATF4-responsive luciferase (21850, Addgene) and 24 h later, cells were treated with the control vehicle or PRMT inhibitors in combination with control or TN for 24 h. The luciferase assay was performed by using dual luciferase assay kit according to manufacturer’s instruction (E4550, Promega).

### Protein analysis

Western blot analyses were performed as previously described^27^. The quantification of protein levels was acquired by the signal intensity analysis using image J (NIH) program and normalized to the loading controls. Immunoprecipitation analysis was carried out as previously described^28^. Briefly, 1 mg of protein lysates in extraction buffer (10 mmol/L Tris-HCl, pH8.0; 150 mmol/L, NaCl; 1 mmol/L EDTA; 1% Triton X-100) containing proteinase inhibitor cocktail (Roche, 1183617001) were immunoprecipitated with 1 μg of primary antibodies conjugated Dynabeads-protein G or protein A complex (20 μl, 50% bead slurry, Invitrogen) for overnight. The primary antibodies used in this study are listed in Table 1s.

### Site-directed mutagenesis

To generate the ATF4 mutants with arginine to lysine switch at arginine residues 239, 244, 257, and 294 of human ATF4, site directed mutagenesis was performed by using QuikChange II XL Site-Directed Mutagenesis Kit (Agilent) followed by manufacture’s instruction. Briefly, mutant human-ATF4 constructs were generated with 0.5 μg of template (pcDNA-Flag-human ATF4, Addgene-plasmid 26114) in sample reaction buffer. After reaction, the synthetized plasmids were transformed into DH5α competent cells after *Dpn1* incubation for 1 h. The primer sequences for mutagenesis are listed in Table 2s.

### RNA analysis

Quantitative real-time polymerase chain reactionq (RT-PCR) and RNA sequencing analysis were performed as previously described^25^. The primer sequences for qRT-PCR are listed in Table 3s For RNA-sequencing analysis, hearts isolated from 2-week-old WT or cKO mice treated vehicle or TN for 16 h were processed to isolated total RNA as previously described^25^. Briefly, mice at postnatal day 14 (*n* = 2 with biological repeats) were intraperitoneally injected with TN (1.5 mg/kg) in 150 mM of dextrose solution (Vehicle, Veh) and 16 h later, hearts were harvested. Total RNAs were extracted with TRizol reagent (Invitrogen, 15596026) and RNA sequencing was carried out with Agilent 2100 bioanalyzer using the RNA 6000 Nano Chip (Agilent Technologies). The analysis for RNA sequencing data was performed by using ExDEGA v1.61 (e-Biogen) and displayed with MeV (v4.9.0) software. The global gene expression was assessed by the reactome with Gene Set Enrichment Analysis (http://software.broadinstitute.org) using MSigDB database v6.1 (>1.3-fold, RC log2 > 2, *P* < 0.05).

### Statistical analysis

Values are means ± SEM or SD as noted. Statistical significance was calculated by paired or unpaired two-tailed Student’s *t* test or analysis of variance test followed by Tukey’s test; differences were considered significant at *P* < 0.05.

## Results

### PRMT1 inhibition aggravates TN-triggered ER stress response in cardiomyocytes

**Fig. 1:**
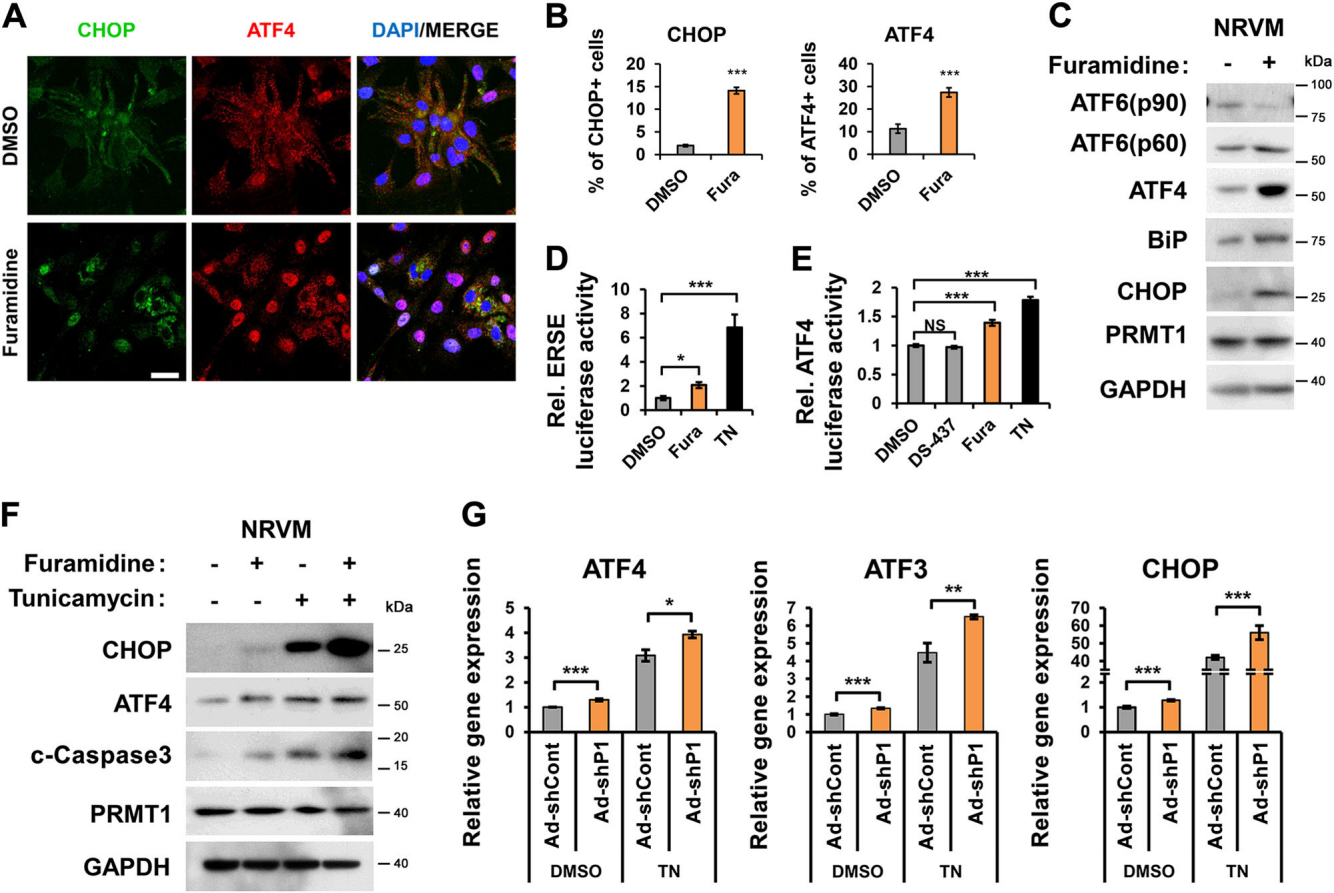
PRMT1 inhibition induces ER stress response in newborn rat ventricular myocytes. **a** Immunostaining for ER stress markers, CHOP (green), and ATF4 (red) in newborn rat ventricular myocytes (NRVMs) treated with vehicle or a PRMT1-specific inhibitor furamidine (Fura, 20 μM) for 24 h. Scale bar = 50 μm. **b** The quantification of CHOP- or ATF4-positive cells from experiments as shown in panel (**a**). Values represent means ± SEM. *n* = 515 cells (Veh); 474 cells (TN) from 5 fields per each experiment with three independent experiments. ^***^P < 0.001. **c** Immunoblotting analysis for the expression of ER stress proteins in NRVMs treated with control or Fura (20 μM) for 24 h. **d** The luciferase reporter analysis driven by the ER stress response element (ERSE) in H9C2 rat cardiomyocytes treated with control, Fura or TN (10 μg/ml) for 24 h. Values represent means of triplicate determinants ±SD. ^*^P < 0.05, ^***^P < 0.001. Experiments were performed at least three times with similar results. **e** The reporter assay with the ATF4-responsive luciferase in H9C2 cells treated with control, Fura, DS-437 (an inhibitor for PRMT5 and 7; 50 μM) and/or TN for 24 h. Values represent means of triplicate determinants ±SD. *n* = 3. ^***^P < 0.001. NS not significant. Experiments were performed at least three times with similar results. **f** Immunoblotting analysis for CHOP, ATF4, cleaved (c)-Caspase3, and PRMT1 in NRVMs treated with vehicle or Fura in combination of control or TN treatment. **g** Relative expression levels for ATF4, ATF3, and CHOP in NRVMs transduced with adenoviral-control shRNA (ad-shCont) or ad-shPRMT1 followed by treatment with vehicle or TN for 24 h. *n* = 3. Error bar shows ±SD. ^*^P < 0.05, ^**^P < 0.01, ^***^P < 0.005.

To examine PRMT1’s role in cardiac ER stress, NRVM, and H9C2 cardiomyocytes were treated with vehicle DMSO or a PRMT1-specific inhibitor (Fura, furamidine) and the expression of ER stress genes was assessed. PRMT1 inhibition greatly increased CHOP- and ATF4-positive cells, compared to the Veh-treated cells (Fig. 1a, b, Fig. 1sA, B). In addition, ATF6 (p60), ATF4, BiP, and CHOP proteins were dramatically upregulated in NRVM and H9C2 cells in response to Fura (Fig. 1c, Fig. 1sC). As expected, the ER stress elements (ERSE)-driven luciferase activities were greatly elevated in H9C2 cells by TN treatment (Fig. 1d). Similarly, PRMT1 inhibition significantly enhanced the ERSE activity. Additionally, ATF4-driven luciferase activities were assessed in H9C2 cells treated with Veh, Fura, TN or a dual PRMT5 and PRMT7 inhibitor, DS-437 as control^29^. The ATF4-luciferase activity was significantly elevated in Fura or TN-treated cells, while the DS-437 treatment did not affect it, relative to control (Fig. 1e). Similar to reporter activities, DS-437 did not affect the expression of ATF4, ATF3, CHOP, and BiP genes, relative to control, while Fura greatly upregulated these genes (Fig. 1sD).

**Fig. 2:**
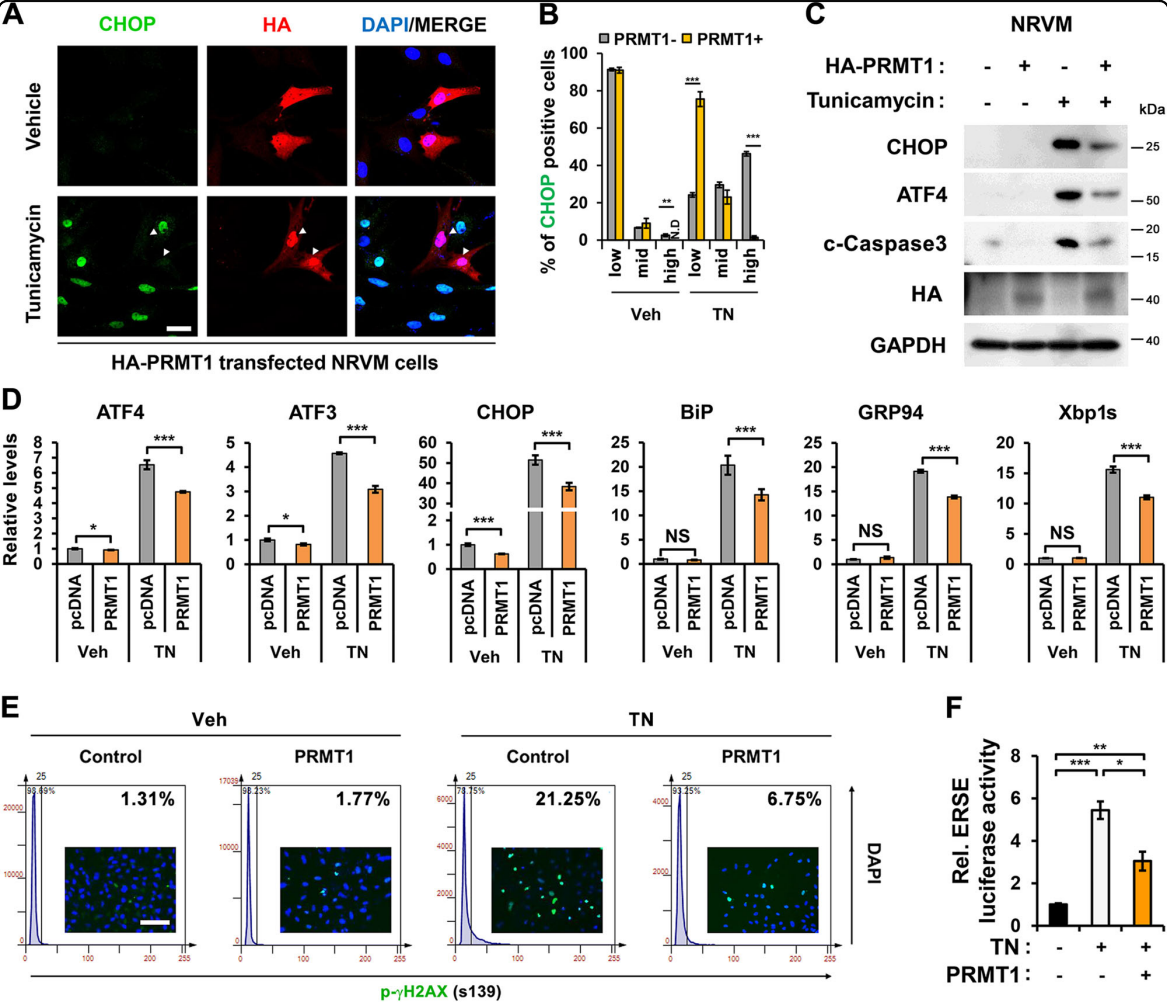
PRMT1 overexpression in rat cardiomyocytes attenuates ER stress and proapoptotic response induced by TN. **a** Representative images of NRVMs treated with DMSO or TN for 24 h, followed by immunostaining with anti-CHOP (green) and anti-HA (red) antibodies. HA-PRMT1-expressing NRVMs are marked with arrowhead. Scale bar = 20 μm. **b** Quantification of the CHOP signal intensity, which is classified as low, medium, and high. Note that PRMT1-overexpressing NRVMs had low levels of CHOP. *n* = 167 cells (Veh); 361 cells (TN) from 4 fields per each experiment with 3 independent experiments. Error bar shows ± SEM. ND not detected, ^**^P < 0.01, ^***^P < 0.005. **c** Immunoblotting analysis for CHOP, ATF4, c-Caspase3 (cleaved-Caspase3), and HA-PRMT1 in control or PRMT1-transfected NRVMs treated with vehicle or TN for 24 h. GAPDH serves as loading control. **d** The mRNA expression for ER stress genes in control or PRMT1-overexpressing NRVMs treated with DMSO or TN. *n* = 3. Error bar shows ±SD. NS not significant, ^*^P < 0.05, ^***^P < 0.005. **e** The TissueFax analysis of p-γH2AX-positive H9C2 cells treated with vehicle or TN for 24 h. Values are determinants of image analysis of 50 fields per each experiment with three independent experiments. **f** The relative activity of the ERSE-luciferase in control or PRMT1-overexpressing H9C2 cells treated with either DMSO or TN for 24 h. *n* = 3. Values are means of triplicate ± SD. ^*^P < 0.05, ^**^P < 0.01 and ^***^P < 0.005.

The effect of Fura on the TN-induced ER stress response and cell death regulators was assessed (Fig. 1sE, F). ATF4, ATF3, CHOP, and BiP were greatly induced by TN and PRMT1 inhibition further elevated their expression except BiP. Fura greatly enhanced transcripts of cell death regulators, Caspase3 and NOXA while DS-437 had no effect. Next, H9C2 cells were treated with Veh or Fura in combination with increasing amounts of TN. The combinatory treatment of TN with Fura greatly elevated the expression of Caspase3 and NOXA (Fig. 1sG, H). In consistence, Fura further enhanced ATF4, CHOP, and cleaved Caspase3 protein levels triggered by TN in both NRVM and H9C2 cells (Fig. 1f, Fig. 1sI, J). In addition, PRMT1-depleted NRVM cells had significantly enhanced ATF4, ATF3, and CHOP levels in both control and TN-treated cells (Fig. 1g, Fig. 1sK). Consistently, TN-treated H9C2 cells for 24 h caused reduction in asymmetric arginine dimethylation of lysates and PRMT1 levels (Fig. 2s), suggesting the importance of PRMT1 in the control of ER stress response. These data suggest that PRMT1 inhibition causes ER stress response in cardiomyocytes and increases susceptibility to cell death.

### PRMT1 overexpression attenuates TN-induced ER stress response in cardiomyocytes

**Fig. 3:**
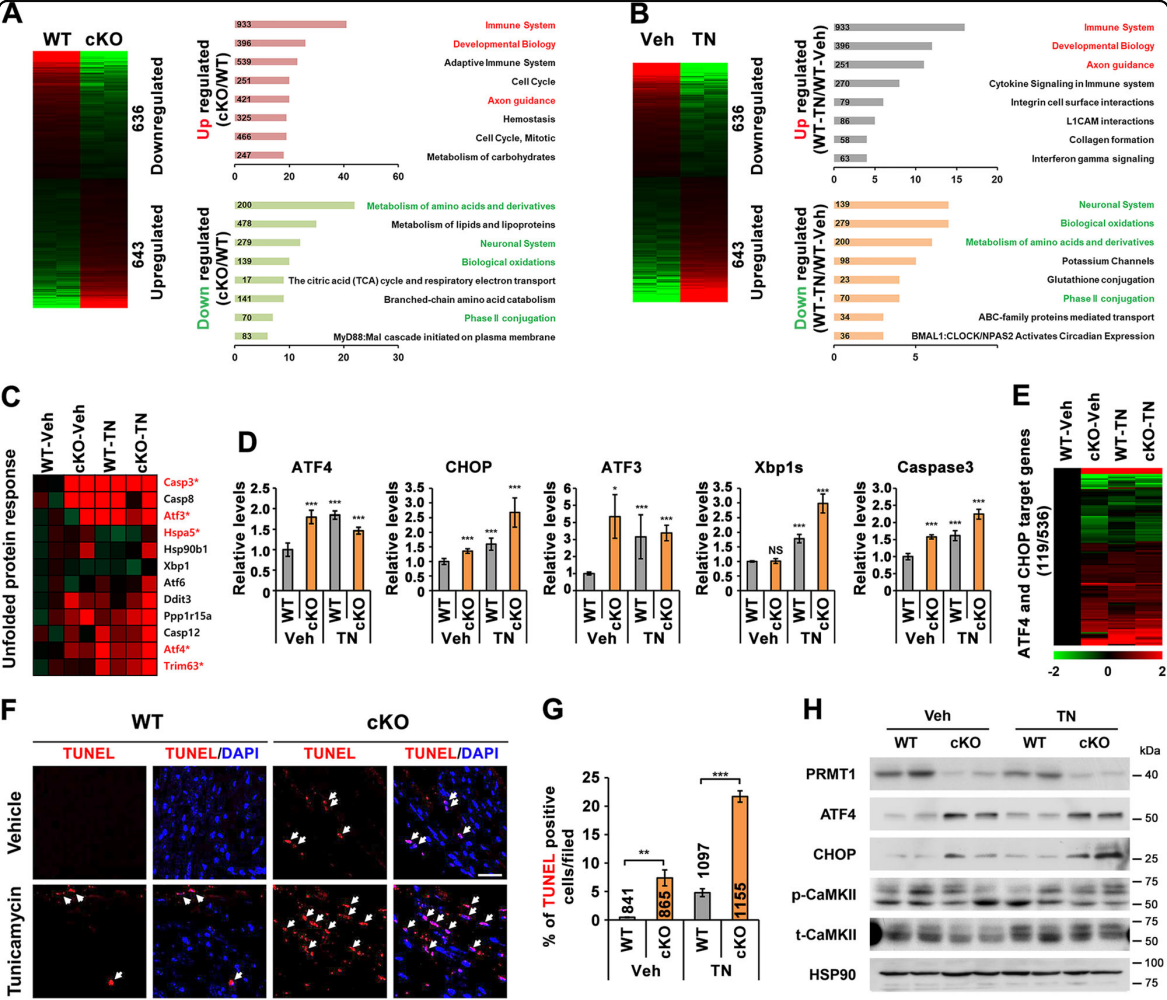
Cardiac-specific PRMT1 deletion elicits endoplasmic reticulum (ER) stress and cardiac cell death. **a**, **b** Comparison of Gene Set Enrichment Analysis for reactome database clusters of genes up- and downregulated between wild type (WT) and cardiac PRMT1-deleted (cKO) or vehicle- and TN-injected hearts. Most significantly affected cellular processes were highlighted. (*n* = 2, with biological repeat, >1.3-fold, normalized with RC log2 > 2, *P* < 0.05). **c** The heat map for RNA expression involved in unfolded protein response genes. Red asterisk marks the significantly altered genes (Cont-Veh vs cKO-Veh, *P < 0.05). **d** The relative mRNA expression levels of ATF4, CHOP, ATF3, Xbp1s, and Caspase3. The average value for WT heart samples is set to 1. Error bar shows ±SD, *n* = 3. NS not significant, ^*^P < 0.05, ^***^P < 0.005. **e** The comparison of gene expression levels for ATF4/CHOP-target genes. Among the 536 ATF4/CHOP-target genes, total 119 genes were significantly changed in vehicle-treated cKO, TN-treated WT, or TN-treated cKO heart samples (>1.3-fold, normalized with RC log2 > 2, *P* < 0.05*)*. **f** Representative confocal images for TUNEL-positive cells (red, marked with white arrow) in WT and cKO hearts treated with vehicle or tunicamycin (TN) for 16 h. Scale bar = 100 μm. **g** Quantification of TUNEL-positive cells in panel (**f**). Counted cell numbers were imbedded in the bar graph. *n* = 6 fields per each experiment with three independent experiments with different samples. Error bar shows ±SEM. ^**^P < 0.01, ^***^P < 0.005. **h** Immunoblot analysis for PRMT1, ATF4, CHOP, an active phosphorylated (p)-CaMKII, and total (t)-CaMKII in WT and cKO hearts treated with vehicle or TN for 16 h.

Next, we examined protective effects of PRMT1 against TN-induced ER stress. Control or Flag-PRMT1-transfected H9C2 cells were treated with vehicle or TN, followed by immunostaing. H9C2 cells with low PRMT1 levels showed strong nuclear ATF4, while cells with high PRMT1 levels exhibited relatively weaker ATF4 expression (Fig. 3sA). As expected, the TN treatment greatly elevated CHOP-positive cells, compared to Veh-treated NRVM and H9C2 cells (Fig. 2a, b, Fig. 3sB, C). PRMT1 levels were inversely correlated with CHOP levels, suggesting a protective role of PRMT1 against TN-induced cell death. PRMT1 overexpression in NRVM cells attenuated ATF4, CHOP and cleaved-Caspase3 levels induced by TN (Fig. 2c). Similarly, PRMT1 overexpressing H9C2 cells showed reduced ATF4 and CHOP protein levels in TN-treated conditions (Fig. 3sD). Moreover, PRMT1-overexpressing NRVM and H9C2 cells expressed substantially less UPR-target genes such as ATF4, ATF3, CHOP, BiP, GRP94, and Xbp1s, compared to TN-treated control cells (Fig. 2d, Fig. 3sE). Further control and PRMT1-overexpressing H9C2 cells were treated with either Veh or TN, followed by immunostaining for p-γH2AX, a DNA damage marker. The TissueFax analysis revealed that control H9C2 cells with TN treatment exhibited 21.25% p-γH2AX positivity, while PRMT1 overexpression attenuated it to 6.75%, in response to TN treatment (Fig. 2e). Furthermore, ERSE luciferase activities induced by TN were significantly attenuated by PRMT1 overexpression in H9C2 cells (Fig. 2f). Taken together, PRMT1 prevents excessive ER stress response of cardiomyocytes upon TN treatment.

### PRMT1-deficient hearts exhibit upregulated ER stress response with elevated ATF4/CHOP axis

Next, we examined ER stress response in mice deleted cardiomyocyte-specific PRMT1 by utilizing Myh6-Cre driven PRMT1 deletion (*PRMT1*^*f/f;Myh6-Cre*^, cKO). The global gene expression of 2-week-old hearts from wildtype *PRMT1*^*f/f*^ (WT) and cKO mice were examined by RNA sequencing analysis (Fig. 3a). The data revealed that genes related with immune system, developmental biology and axon guidance were significantly upregulated while genes involved in amino acids metabolism, neuronal system, biological oxidation and phase II conjugation were significantly downregulated in cKO hearts, compared to WT hearts. In addition, 2-week-old WT mice were injected with vehicle or TN (1.5 mg/kg body weight) and 16 h later, hearts were harvested, followed by RNA sequencing. As shown in Fig. 3a, b, the reactome of cKO hearts was strikingly similar to TN-treated control hearts, suggesting that PRMT1 deficiency augments ER stress response in hearts.

The detailed transcriptome analysis suggested that cKO hearts had elevated levels of UPR genes, similarly to TN-treated control hearts (Fig. 3c). Caspase3, ATF3, Hspa5, ATF4, and Trim63 were significantly changed in cKO hearts regardless of TN treatment. However, the level of ATF6 and its target Xbp1 were not significantly altered in cKO hearts, relative to control. The qRT-PCR analysis further confirmed that ATF4 and its target genes, CHOP and ATF3, were significantly increased in both Veh- and TN-cKO hearts, while Xbp1s was only elevated in TN-treated samples but not in cKO without TN treatment (Fig. 3d). Consistently, Caspase3 was increased in cKO hearts, similarly to TN-treated control hearts. Among the previously proposed 536 target genes of ATF4 and CHOP^30^, the expression of 119 genes were changed in both Veh-cKO and TN-hearts (Fig. 3e).

To assess myocardial cell death, the TUNEL assay with control and cKO 2-week-old hearts treated with vehicle or TN were performed (Fig. 3f, g). The vehicle-treated WT (Veh-WT) hearts did not show cell death but the TN-induced cell death with about 5% TUNEL-positive cells. Interestingly, vehicle-treated cKO (Veh-cKO) hearts exhibited roughly 8% TUNEL-positive cells, which was further increased to roughly 22% upon TN treatment. These data suggest that PRMT1 deficiency results in cardiac cell death and aggravates ER stress-induced cell death. Consistently, Veh-cKO hearts had greatly elevated ATF4 and CHOP levels, compared to the Veh-control hearts (Fig. 3h). The TN treatment upregulated ATF4 and CHOP proteins in both control and cKO hearts. Previously, we showed that CaMKII hyperactivation was observed in PRMT1-deficient hearts starting 4-weeks and CaMKII repressions prevented hypertrophic responses and cardiac dysfunction in cKO mice^21^. Thus, we examined the activation state of CaMKII in 2-week-old WT and cKO hearts treated with vehicle or TN. There was no obvious alteration in p-CaMKII levels correlating with ER stress. These data suggest that ER stress deregulation occurs prior to cardiac remodeling and CaMKII dysregulation in cKO hearts. Collectively, PRMT1 deficiency in hearts causes deregulation of ATF4/CHOP pathway, leading to cardiac cell death.

### ATF4 depletion attenuates ER stress response triggered by PRMT1 inhibition

**Fig. 4:**
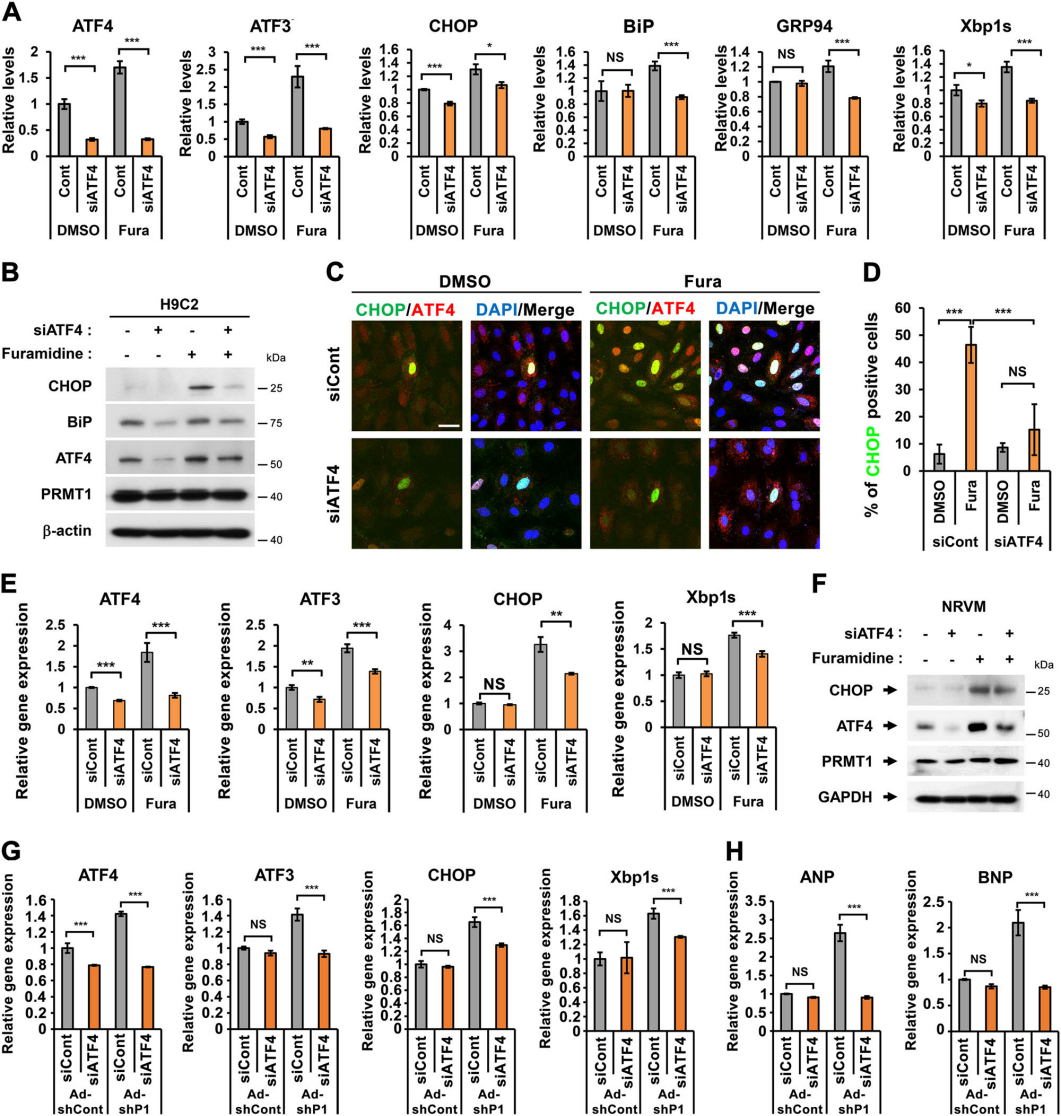
The ER stress response triggered by PRMT1 inhibition is attenuated by ATF4 knockdown in rat cardiomyocytes. **a** The expression of ATF4, ATF3, CHOP, BiP, GRP94, and Xbp1s in control or siATF4-transfected H9C2 cells followed by the treatment with control DMSO or Fura for 24 h. *n* = 3. Error bar shows ±SD. NS not significant, ^*^P < 0.05, ^***^P < 0.005. **b** Immunoblot analysis for ATF4, BiP, CHOP, and PRMT1. β-actin were used as loading control. **c** Representative confo**c**al images for CHOP and ATF4 in control or siATF4-transfected H9C2 cells treated with control DMSO or Fura for 24 h. *n* = 3 fields per each experiment with three independent experiments. Scale bar = 20 μm. **d** Quantification of CHOP-positive H9C2 cells in panel (**c**). Values are determinants of 12 fields ±SD. NS not significant, ***P < 0.005. Experiments are repeated three times with similar results. **e** The relative gene expression of ATF4, ATF3, CHOP, and Xbp1s in control or siATF4-transfected NRVMs with DMSO or Fura treatment for 24 h. *n* = 3. Error bar shows ±SD. NS not significant, ^**^P < 0.01, ^***^P < 0.005. **f** Immunoblotting analysis for CHOP, ATF4, and PRMT1 in control or siATF4-transfected NRVMs with DMSO or Fura treatment for 24 h. **g**, **h** The mRNA expression of ATF4, ATF3, CHOP, Xbp1s, and hypertrophic gene ANP and BNP in NRVM cells transduced with Ad-shCont or Ad-shPRMT1 along with transfection with control scrambled or siATF4. *n* = 3. Error bar shows ±SD. NS not significant, ^***^P < 0.005.

H9C2 cells were transfected with control or ATF4 siRNA (siATF4) and treated with Veh or Fura. ATF4 depletion in H9C2 cells significantly reduced the expression of ATF3, CHOP, GRP78, GRP94, and Xbp1s induced by Fura treatment. In addition, ATF4-depleted H9C2 cells had greatly reduced ATF4 and BiP proteins and CHOP was barely detectable (Fig. 4a, b). Fura-treated control cells greatly enhanced ATF4 and CHOP protein levels. In contrast, ATF4 depletion abrogated the Fura-induced increase in ATF4 and CHOP protein levels. Immunostaining analysis confirmed that the Fura treatment-induced increase in CHOP-positive H9C2 cells was alleviated by ATF4 knockdown (Fig. 4c, d). Similar to H9C2 cells, ATF4 depletion in NRVM cells significantly reduced ATF3, CHOP, and Xbp1s induced by Fura treatment (Fig. 4e). Moreover, ATF4 depletion led to the attenuated CHOP increase in NRVM cells (Fig. 4f). Consistently, PRMT1-depleted NRVM cells elevated levels of ATF4, ATF3, CHOP, and Xbp1s, while ATF4 depletion abrogated this increase (Fig. 4g). The increased expression of ANP and BNP in PRMT1-depleted NRVMs was repressed by ATF4 depletion (Fig. 4h), suggesting for a potential role of ER stress in cardiac remodeling. Collectively, ATF4 is required for PRMT1-mediated ER stress regulation in cardiomyocytes.

### The arginine 239 to lysine mutation in ATF4 exacerbates ER stress response and cell death in cardiomyocytes

**Fig. 5:**
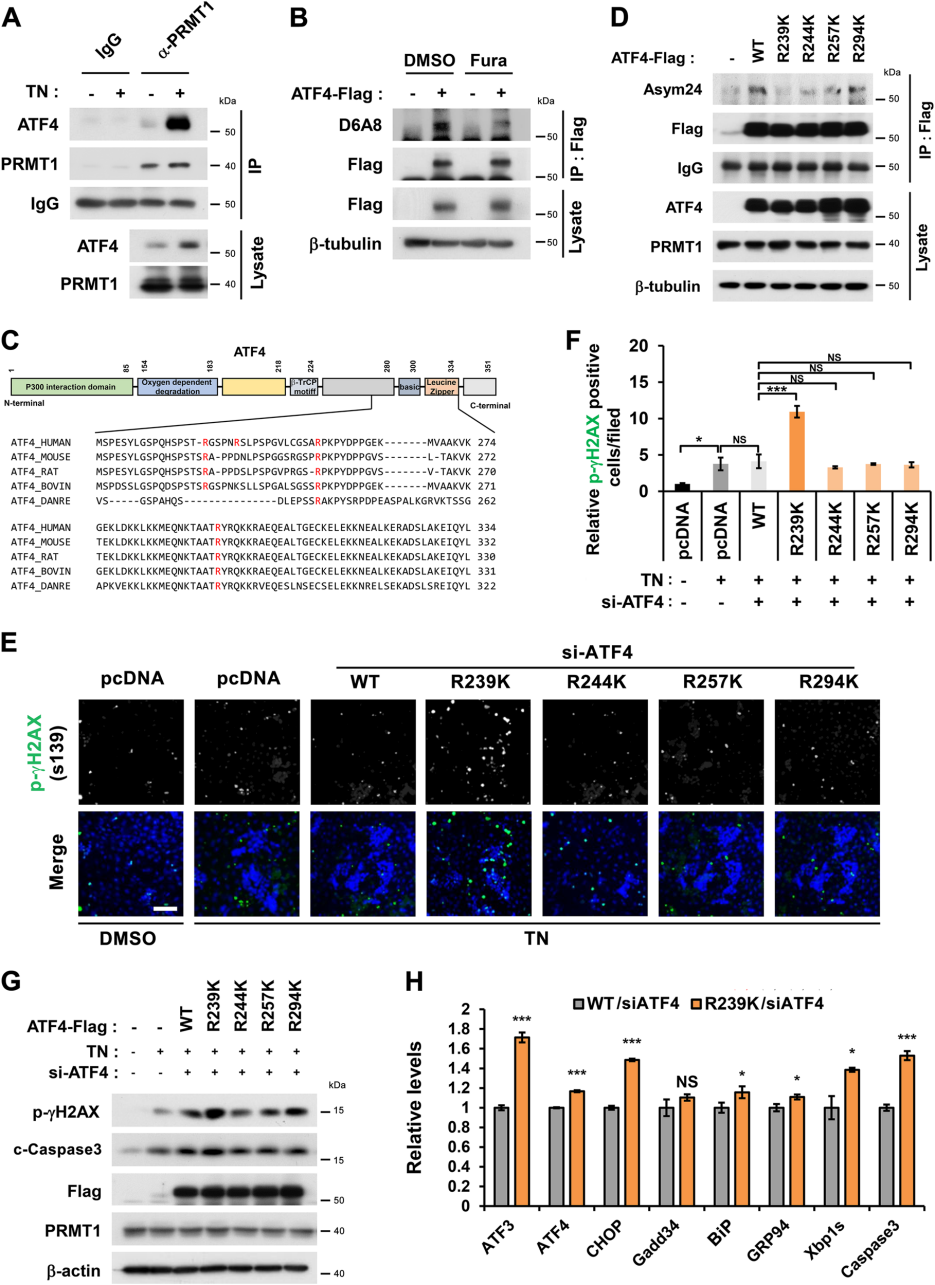
Methylation of ATF4 on arginine 239 by PRMT1 is critical for the suppression of ER stress and cell death induced by TN treatment. **a** Co-immunoprecipitation for endogenous ATF4 and PRMT1 after the treatment with TN for 24 h. **b** Immunoblot analysis for asymmetric arginine demethylation of ATF4 with D6A8 antibody in control DMSO or Fura-treated HEK293T cells. **c** Schematic illustration for the sequence homology in the region of ATF4 spanning from aa223 to aa334 (human ATF4) from various species and the potential PRMT1 target arginine residues are labeled red. Note that arginine 244 is only found in human ATF4. **d** Immunoblotting for asymmetric dimethylation with ATF4 WT and mutants. HEK293T cells expressing Flag-tagged ATF4 WT or four mutants with arginine to lysine switch (R239K, R244K, R257K, and R294K) were subjected to immunoprecipitation with Flag antibody followed by immunoblotting with Asym24 antibody. **e** Representative images for p-γH2AX-positive H9C2 cells expressing indicated ATF4 proteins in response to TN treatment. Scale bar = 300 μm. **f** Quantification of p-γH2AX positive cells from three independent experiments shown in panel e. Values are means of 12 determinants ± SEM. (*n* = 12), NS = not significant, ^*^P < 0.05, ^***^P < 0.005. **g** Immunoblot analysis for p-γH2AX, c-Caspase3, and PRMT1 in addition to ectopically expressed Flag-ATF4 WT and RK mutants. β-actin serves as loading control. **h** qRT-PCR analysis for ATF3, ATF4, CHOP, Gadd34, BiP, GRP94, Xbp1s, and Caspase3 in ATF4-depleted H9C2 cells transfected with ATF4 WT or R239K mutant and treated with TN for 8 h. Values are means of three determinants ±SD. (*n* = 3). NS not significant, ^*^P < 0.05, ^***^P < 0.005.

To investigate PRMT1’s role in ATF4 activity control, the interaction between PRMT1 and ATF4 was verified. PRMT1 and ATF4 were coprecipitated in a reciprocal manner (Fig. 4s). The TN treatment greatly elevated ATF4 levels coprecipitated with PRMT1 (Fig. 5a), suggesting the enhanced interaction between ATF4 and PRMT1 in response to ER stress. To examine ATF4 methylation by PRMT1, Flag-tagged ATF4 was overexpressed and immunoprecipitated with Flag antibody, followed by immunoblotting with anti-asymmetric methyl-arginine antibody^24^ (Fig. 5b). ATF4 was indeed arginine-methylated and PRMT1 inhibition greatly reduced methylated ATF4 levels.

To examine the role of ATF4 methylation in the ER-stress mediated cell death, we have generated four arginine-to-lysine mutants. In addition to R239 which has been previously reported, the sequence analysis by GPS-methyl-group specific predictor software predicted R244, R257, and R294 of human ATF4 as potential methylation sites (red) (Fig. 5c). Consistent with the previous report^16^, a lysine switch of ATF4 at arginine 239, R239K/ATF4 showed significantly reduced asymmetric dimethylation assessed by Asym24 antibody, compared to WT or other mutants. Therefore, the R239 of ATF4 is a target site of PRMT1 (Fig. 5d). To investigate the functional significance of ATF4 methylation, H9C2 cells were transfected with control, WT/ATF4 or 4 different arginine-to-lysine ATF4 mutants (R239K, R244K, R257K, or R294K), followed by TN treatment and cell death analysis (Fig. 5s). Control pcDNA or ATF4 proteins (WT, R244K, R257K, or R294K) expressing H9C2 cells showed roughly 50–60% live cells without nuclear fragmentation in response to TN, while R239K/ATF4-expressing H9C2 cells displayed a greatly enhanced proportion of dying cells with nuclear fragmentation (Fig. 5sA, B). In addition, these cells were subjected to immunostaining for CHOP (Fig. 5sC, D). Similarly to nuclear fragmentation, R239K/ATF4-expressing cells showed significantly elevated CHOP signal intensity, compared to other cells.

**Fig. 6:**
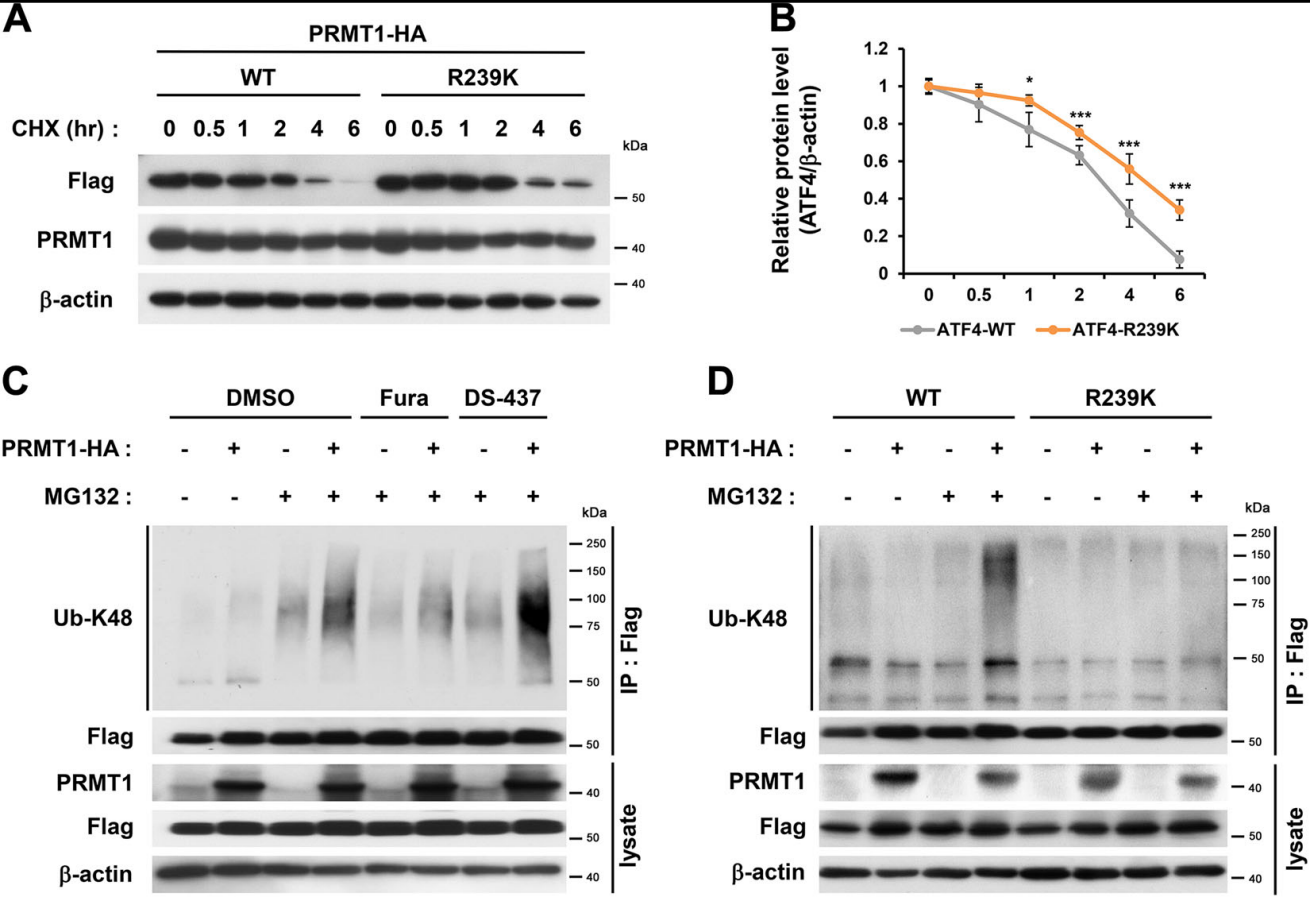
Arginine methylation of ATF4 by PRMT1 regulates ATF4 protein stability. **a** Immunoblot analysis for Flag-tagged ATF4-WT or R239K in HEK293T cells treated with cycloheximide (CHX, 10 μg/ml) for indicated hours. **b** The quantification of the relative Flag-ATF4 protein levels from experiments shown in panel (**a**). The signal intensities of Flag-ATF4 WT and R239K are normalized to β-actin loading control. The average values of WT at time point 0 is set to 1. Values are means of three independent determinants ±SEM. *n* = 3, *P *<* 0.05. ***P < 0.005. **c** Immunoblot analysis of ubiquitinated Flag-ATF4 protein in HEK293T cells transfected with expression vectors for Flag-ATF4 and ubiquitin along with control or PRMT1-HA. Cells were treated with control DMSO, Fura (10 μM) or DS-437 (50 μM) for 24 h. Four hour prior to harvest, cells were treated with MG132 (10 μM) to prevent proteasomal degradation. **d** Immunoblot analysis of ubiquitinated Flag-ATF4 proteins. HEK293T cells were cotransfected with expression vectors for ubiquitin in combination of ATF4 WT or R239K along with control or PRMT1-HA. Cells were treated with vehicle or a proteasome inhibitor MG132 (10 μM) for 4 h, followed by immunoprecipitation with Flag antibody and immunoblotting with Ub-K48 antibody to detect ubiquitinated ATF4 proteins.

Next, control or ATF4-depleted H9C2 cells were transfected with WT or ATF4 mutants, followed by Veh or TN treatment for 24 h and p-γH2AX immunostaining (Fig. 5e, f). R239K/ATF4 led to greatly elevated levels of p-γH2AX-positivity, compared to WT and other mutants. TN treatment enhanced the level of cleaved Caspase3 and p-γH2AX, and ATF4 overexpression further elevated these protein levels (Fig. 5g). Consistently, R239K mutant expression led to the strongest expression of cleaved Caspase3 and p-γH2AX in response to TN, likely contributing to cardiomyocyte death. Further, the activity of WT/ATF4 or R239K/ATF4 in response to TN treatment for 8 h was assessed by qRT-PCR (Fig. 5h). The level of ATF3, CHOP, Caspase3, and Xbp1s was significantly enhanced in R239K/ATF4-expressing cells, compared to the WT/ATF4-expressing cells. In addition, the effect of PRMT1 overexpression on ATF4 activity was assessed (Fig. 6s). Reintroduction of WT/ATF4 or R239K/ATF4 in ATF4-depleted H9C2 cells significantly elevated ATF4-reporter activities in response to TN. PRMT1 overexpression almost fully blunted WT/ATF4-mediated activities while it failed do so in R239K/ATF4-expressing cells. Similarly, PRMT1 overexpression decreased levels of ATF3, CHOP and Caspase3 by in ATF4-expressing cells however R239K/ATF4-expressing cells showed significant resistance to PRMT1, suggesting a suppressive role for PRMT1-mediated arginine methylation in ATF4 activity.

### PRMT1 regulates ATF4 protein stability though methylation of R239

To assess the ATF4 protein stability, H9C2 cardiomyocytes were transfected with PRMT1-HA and WT/ATF4 or R239K/ATF4. 48 h later, cells were treated with cycloheximide (CHX) for indicated hours. R239K/ATF4 proteins degraded slower than WT/ATF4 proteins (Fig. 6a, b). At 6 h-CHX treatment, less than 10% of WT/ATF4 proteins remained, but about 40% of R239K/ATF4 proteins were still maintained. H9C2 cells were transfected with Flag-ATF4 and Ubiquitin in a combination with control or PRMT1-HA, followed by the treatment with vehicle or the proteasome inhibitor MG132 in combination with vehicle and PRMT inhibitors (Fura or DS-437) (Fig. 6c). Ub-K48-WT/ATF4 levels in PRMT1-overexpressing cells is increased by MG132 treatment for 4 h and Fura reduced Ub-K48-WT/ATF4 levels in both control and PRMT1-overexpressing cells. In contrast, DS-437 failed to suppress the ATF4-ubiquitination induced by PRMT1. In addition, PRMT1 overexpression elevated Ub-K48-WT/ATF4 levels, while the R239K mutant did not show any change in ubiquitination state, regardless of PRMT1 levels (Fig. 6d). Taken together, our data suggest that PRMT1 deficiency causes deregulated ATF4/CHOP pathway and cardiac cell death. PRMT1 regulates ATF4 stability through methylation of R239. In conclusion, PRMT1 plays a protective role against ER stress-mediated cell death in myocardium.

## Discussion

Accumulating evidences suggest a critical role of ER stress in development of cardiac diseases, thus the modulation of ER stress responses is an attractive therapeutic strategy to intervene cardiovascular and heart diseases^2,31,32^. In this study, we demonstrate the importance of PRMT1 in suppression of ER stress. We show that PRMT1-deleted cardiomyocytes and hearts exhibit enhanced ER stress response genes, especially ATF4-target genes. Conversely, PRMT1 overexpression in cardiomyocytes decreases ATF4/CHOP cell death pathway in response to TN treatment. These data suggest the preventive role of PRMT1 against ER-stress induced cardiac cell death. Consistently, PRMT1-deficient hearts exhibit abnormal cell death and DNA damage which is further enhanced by TN treatment. We also provide evidence that PRMT1 suppresses the ER-stress induced cell death by modulating ATF4 methylation on R239. Consistently, the expression of R239K/ATF4 in cardiomyocytes results in enhanced levels of p-γH2AX and cleaved-Caspase3, compared to WT/ATF4 or other RK mutants.

Based on current data, we propose that PRMT1 is critical for the control of ER stress and cardiac function by ATF4 methylation. The RNA sequencing analysis revealed that genes linked with amino acid metabolism and oxidative stress, well known ER stress inducing pathways, are dysregulated in PRMT1 cKO hearts. Thus, it is conceivable that perturbed amino acid metabolism and oxidative stress in PRMT1-deficient hearts might be initiating ER stress. ER stress response is known to inhibit general protein synthesis and increase protein folding and degradation to reestablish ER homeostasis, ultimately leading to cell survival^33–35^. However, PRMT1 deficiency and inhibition cause deregulation of ATF4 and ER stress response. Therefore, PRMT1 might be critical to maintain metabolic health of cardiac cells and manage the stress-induced repair pathways. The exact mechanism leading to perturbed amino acid metabolism and oxidative stress in PRMT1 null hearts is currently unclear. Considering the functional demands that heart needs to meet for the postnatal adaptation and maturation, various mechanisms accompanied by basal oxidative stress and ER stress are activated that might be properly controlled under the threshold levels to maintain cardiac function^36^. Thus, it is tempting to speculate that PRMT1 might act as a safeguard to maintain the ER stress under the threshold levels.

ER stress has been implicated in multiple cardiovascular diseases, including dilated cardiomyopathy^7^. However, considering that PRMT1 is the major cellular PRMT and heart failure of cKO mice rapidly progressed, we do not rule out the involvement of other pathways. An additional mechanism for ER stress initiation in cKO hearts might be the perturbed Ca^2+^ homeostasis in cardiomyocytes. Previously, we have demonstrated that PRMT1 is critical for suppression of CaMKII through methylation^21^. Interestingly, however, CaMKII hyperactivation was not observed in 2-week-old PRMT1-deficient hearts when ER stress response is elevated. Thus, we concluded that CaMKII hyperactivation is not the initiating factor for ER stress in PRMT1-deficient hearts. The rapidly progressing cardiomyopathy and heart failure observed in PRMT1 cKO within 8-weeks after birth likely is due to its role in the control of critical regulators for cardiac function such as CaMKII and ATF4 that are directly contributing to ER stress and heart failure. The fact that ER stress was observed prior to cardiac remodeling gene expression and CaMKII hyperactivation in cKO hearts underlines the importance of PRMT1 in suppression of ER stress to maintain cardiac function.

As previously reported, 6-week-old PRMT1-deficient hearts exhibited abnormal alternative splicing, likely contributing to contractile dysfunction and cardiomyopathy^22^. Thus PRMT1 seems to be critical for alternative splicing during postnatal 4-weeks involved in cardiac maturation for the adult life^37^. Currently, it is unclear the relationship between the aberrant alternative splicing and ER stress in PRMT1-deficient hearts. The comparison of the global gene profile between 2-week-old cKO from the current study and 6-week-old cKO from the study by Murata et al.^22^ revealed about 10% overlapping global genes (Dataset 1). Among 958 downregulated genes in 6-week-old cKO hearts, 150 genes were altered in 2-week-old cKO hearts (1.5-fold, Normalized RC log2 > 2) with 129 downregulated genes and 21 upregulated genes. In the close examination, 3 genes (Itgb6, Cacna2d3, and Cacna1c) were downregulated in 2-week-old cKO hearts, compared to 15 genes downregulated in KEGG_dilated cardiomyopathy pathway in 6-week-old cKO. In 2-week-old cKO hearts, only 7 genes among the 92 KEGG_dilated cardiomyopathy related genes were significantly altered (*p* value = 4.99e−3, *q*-value = 1.75e−2). Thus, this difference in gene expression profile is likely due to the severity of cardiomyopathy phenotype observed in 6-week-old cKO hearts compared to that in 2-week-old cKO hearts. In the close examination of the expression of RNA binding proteins implicated in alternative splicing, only Rbfox1 expression was greatly decreased in 2-week-old cKO hearts. Unlike the severe cKO phenotypes, mice null for cardiac-specific Rbfox1 did not show any defects in cardiac function at 2 months of age and the mildly decreased cardiac function was observed at 6 month of age^38^, suggesting that Rbfox1 is not a cause of cell death observed in 2-week-old cKO hearts. Thus, ER stress deregulation is likely the causative factor for cell death in cKO hearts.

The methylation of ATF4 on R239 by PRMT1 has been previously determined by Yuniati et al.^16^, which is in line with our current result. However, they proposed that PRMT1 is recruited to a tumor suppressor BTG1 and ATF4 and methylates ATF4 leading to induction of cell stress-related genes^16^. The basis for this discrepancy is currently unclear, however, one of the obvious differences might be the cell systems used in two studies. Yuniati et al. has used mostly BTG1-mutant mouse embryonic fibroblasts to assess BTG1-regulated ATF4 activity in response to various metabolic stresses. It is unclear whether BTG1 is involved in ER stress response in cardiac tissues or whether PRMT1 is important for metabolic stress response in cardiac tissues. Further studies are required to answer these questions. Regardless, our study demonstrates the critical role of PRMT1 in suppression of ATF4/CHOP pathway to prevent cardiac cell death.

## Supplementary information

Legends of supplemenatal figures

Figure 1s

Figure 2s

Figure 3s

Figure 4s

Figure 5s

Figure 6s

Supplementary Tables

DATASET1
