# Correction: PRMT1 suppresses ATF4-mediated endoplasmic reticulum response in cardiomyocytes

**DOI:** 10.1038/s41419-020-2373-8

**Published:** 2020-03-23

**Authors:** Myong-Ho Jeong, Hyeon-Ju Jeong, Byeong-Yun Ahn, Jung-Hoon Pyun, Ilmin Kwon, Hana Cho, Jong-Sun Kang

**Affiliations:** 1 Department of Molecular Cell Biology, Sungkyunkwan University, Suwon, Republic of Korea; 2 Single Cell Network Research Center, Sungkyunkwan University, Suwon, Republic of Korea; 3 Division of Cardiovascular Diseases, Center for Biomedical Sciences, National Institute of Health, Cheongju, Chungbuk Republic of Korea; 4 Department of Anatomy and Cell Biology, Sungkyunkwan University, Suwon, Republic of Korea; 5 Department of Physiology, Sungkyunkwan University, Suwon, Republic of Korea

**Keywords:** Cell death, Heart failure

**Correction to: Cell Death & Disease**

10.1038/s41419-019-2147-3 published online 02 December 2019

Following publication of this article, the authors found a numerical notation error in Fig. 3B. 636 should have read 240, and 643 should have read 263.This has been corrected in the PDF and HTML versions.

