# Supplementary material for: PRMT1 suppresses ATF4-mediated endoplasmic reticulum response in cardiomyocytes
Source: Cell Death Dis. 2019 Dec 2;10(12):903. doi: 10.1038/s41419-019-2147-3 (PMC6885520; doi:10.1038/s41419-019-2147-3)
Supplement: Supplementary file 1 — Legends of supplemenatal figures [file 41419_2019_2147_MOESM1_ESM.docx]

**Supplementary Figure Legends**

**Figure 1s. PRMT1 inhibition exacerbates ER stress response caused by TN in rat cardiomyocytes.**

(**A**) Immunostaining of ER stress markers, CHOP (green) and ATF4 (red) for H9C2 cardiomyocyte with the treatment of vehicle or a PRMT1-specific inhibitor Furamidine (Fura, 20μM) for 24 hours. Scale bar = 50μm. (**B**) Quantification of CHOP or ATF4 positive cells from experiments similarly performed as shown in panel A. n=294 cells (DMSO); 188 cells (Fura) from 4 fields per each experiment with three independent experiments. Values represent means ± SEM. N=3. *^***^P<0.001*. (**C**) Immunoblotting analysis for ER stress pathway proteins from H9C2 cells treated with control or Fura (20μM) for 24 hours. (**D**) qRT-PCR analysis for ER stress response genes in H9C2 cells treated with Fura or DS-437 for 48 hours. n=3. Error bar shows ± SD. *^**^P<0.01, ^***^P<0.005*. NS= not significant. (**E**) qRT-PCR analysis for ER stress response genes in H9C2 cells treated with TN for 24 hours prior to the treatment with vehicle or Fura for 48 hours. *^*^P<0.05, ^***^P<0.005*. Error bar shows ± SD. n=3. NS=not significant. (**F**) qRT-PCR analysis for cell death markers, Caspase3 and NOXA, in H9C2 cells treated with Fura or DS-437 for 48 hours. n=3. Error bar shows ± SD. *^**^P<0.01, ^***^P<0.005*. NS=not significant. (**G-H**) Relative expression levels for Caspase3 and NOXA in H9C2 cells treated with vehicle or Fura plus TN with increasing concentration. n=3. Error bar shows ± SD. *^*^P<0.05, ^**^P<0.01, ^***^P<0.005*. (**I**) Immunoblotting analysis for CHOP, ATF4, cleaved (c)-Caspase3 and PRMT1 in H9C2 cells treated with vehicle, Fura, plus TN with increasing amounts. (**J**) Quantification analysis for the relative protein levels of CHOP, ATF4 and c-Caspase3 from experiments shown in panel I. n=3. Error bar shows ± SD. *^***^P<0.005*. NS=not significant. (**K**) Immunoblotting analysis for adenovirus mediated PRMT1 depletion in NRVM cells. β-actin is served as loading control.

**Figure 2s. The TN treatment blunts the levels of asymmetric arginine dimethylation and PRMT1 protein in H9C2 cardiomyocytes.**

(**A**) Immunoblotting analysis of H9C2 cells treated with control and TN ranging from 2.5 to 10 μg/ml for 24 hours with antibodies recognizing the asymmetrically dimethylated arginine or PRMT1.

**Figure 3s. PRMT1 overexpression in cardiomyocytes attenuates ER stress response triggered by TN.**

(**A**) Immunostaining for PRMT1 (green) and ATF4 (red) in control or Flag-PRMT1 transfected H9C2 cells treated with TN for 24 hours. Scale bar = 20μm. (**B**) Representative images of H9C2 cells treated with DMSO or TN for 24hours, followed by immunostaining for CHOP (green) and Flag-PRMT1 (red) expression. Flag-PRMT1 expressing H9C2 cells are marked with arrowhead. Scale bar = 20μm. (**C**) Quantification of CHOP signal intensities classified as low, medium and high. Note that PRMT1 overexpressing H9C2 cells exhibit low levels of CHOP proteins. n=279 cells (Veh); 249 cells (TN) from 4 fields per each experiment with three independent experiments. Error bar shows ± SEM. *^***^P<0.005*. ND=not detected. (**D**) Immunoblotting analysis for PRMT1, ATF4 and CHOP in TN-treated H9C2 cells transfected with control or PRMT1. β-actin serves as loading control. (**E**) qRT-PCR analysis for ER stress genes in control or PRMT1 overexpressing H9C2 cells treated with DMSO or TN. n=3. Error bar shows ± SD. NS=not significant. *^***^P<0.005*.

**Figure 4s.** **PRMT1 forms complexes with ATF4.**

(**A, B**) Immunoblot analysis for interaction between PRMT1 and ATF4 in HEK293T cells expressing ATF4-Flag and PRMT1-HA.

**Figure 5s. Methylation of ATF4 at R239 by PRMT1 is important for suppression of cardiomyocyte death triggered by TN treatment.**

(**A**) Representative images for H9C2 cells expressing control or various ATF4 proteins treated with TN for 24 hours. Fragmented nuclei are labelled with red pseudo-color. Scale bar = 20μm (**B**) Quantification of cells with fragmented nuclei from experiments shown in panel A. Total counted cell numbers were imbedded in the bar graph. Values represent determinants from 12 fields per each experiment with three independent experiments. Error bar shows ± SEM. *^*^P<0.05*, *^***^P<0.005* vs pcDNA, *^#^P<0.05*, *^###^P<0.005* vs WT. (**C**) Immunostaining for CHOP positive H9C2 cells transfected with control, ATF4 WT or mutant and treated with TN for 24hrs. Scale bar = 20μm (**D**) Quantification of relative CHOP signal intensity normalized by DAPI signal from experiments shown in panel C. Values are determinants of 12 fields per each experiment from three independent experiments. Error bar shows ± SEM. *^***^P<0.005*.

**Figure 6s. PRMT1 overexpression fully suppresses WT/ATF4-mediated activity while R239K/ATF4-mediated activity is only partially affected.**

(**A**) The reporter assay with the ATF4-responsive luciferase in H9C2 cells cotransfected with siATF4, WT/ATF4 or R239K/ATF4 mutant and PRMT1 treated with TN (2.5μg/ml) for 16 hours. Values represent means of triplicate determinants ± SD. n=4. Experiments were repeated with similar results. *^*^P<0.05*, *^**^P<0.01*, *^***^P<0.005* vs scrambled siRNA plus control vectors, *^##^P<0.01*, *^###^P<0.005* vs siATF4 plus WT/ATF4, *^$$$^P<0.005* vs siATF4 plus WT/ATF4 and PRMT1-HA. (B) (**h**) qRT-PCR analysis for ATF3, CHOP, and Caspase3 in ATF4-depleted H9C2 cells transfected with WT/ATF4 or R239K/ATF4 mutant and control or PRMT1 treated with TN (10μg/ml) for 24 hours. Values are means of three determinants ± SD. (n=3). NS=not significant, *^**^P<0.01, ^***^P<0.005.*
