# Supplementary figures and images for: PRMT1 suppresses ATF4-mediated endoplasmic reticulum response in cardiomyocytes
Source: Cell Death Dis. 2019 Dec 2;10(12):903. doi: 10.1038/s41419-019-2147-3 (PMC6885520; doi:10.1038/s41419-019-2147-3)

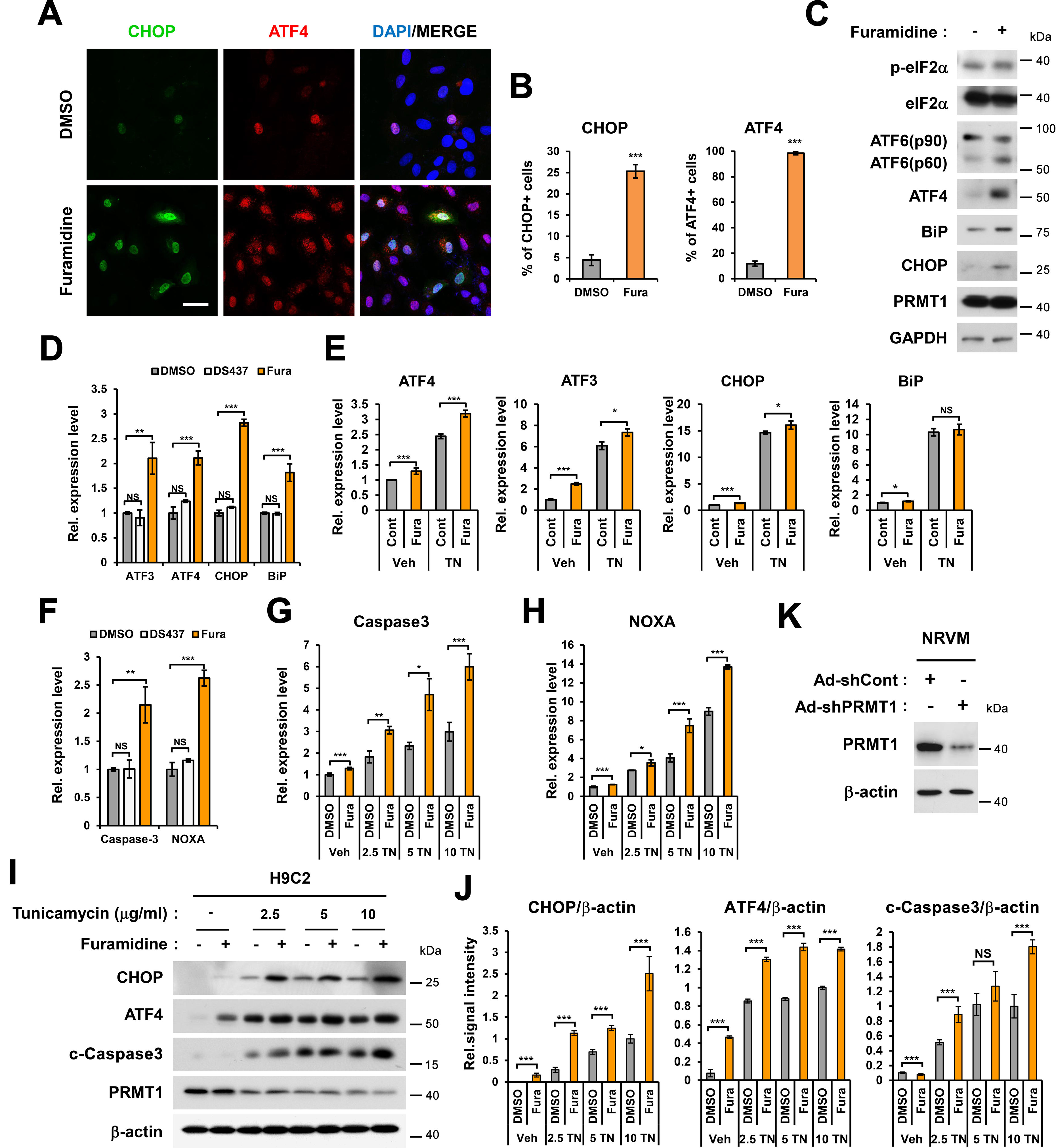

Supplement: Supplementary file 2 — Figure 1s [file 41419_2019_2147_MOESM2_ESM.tif]

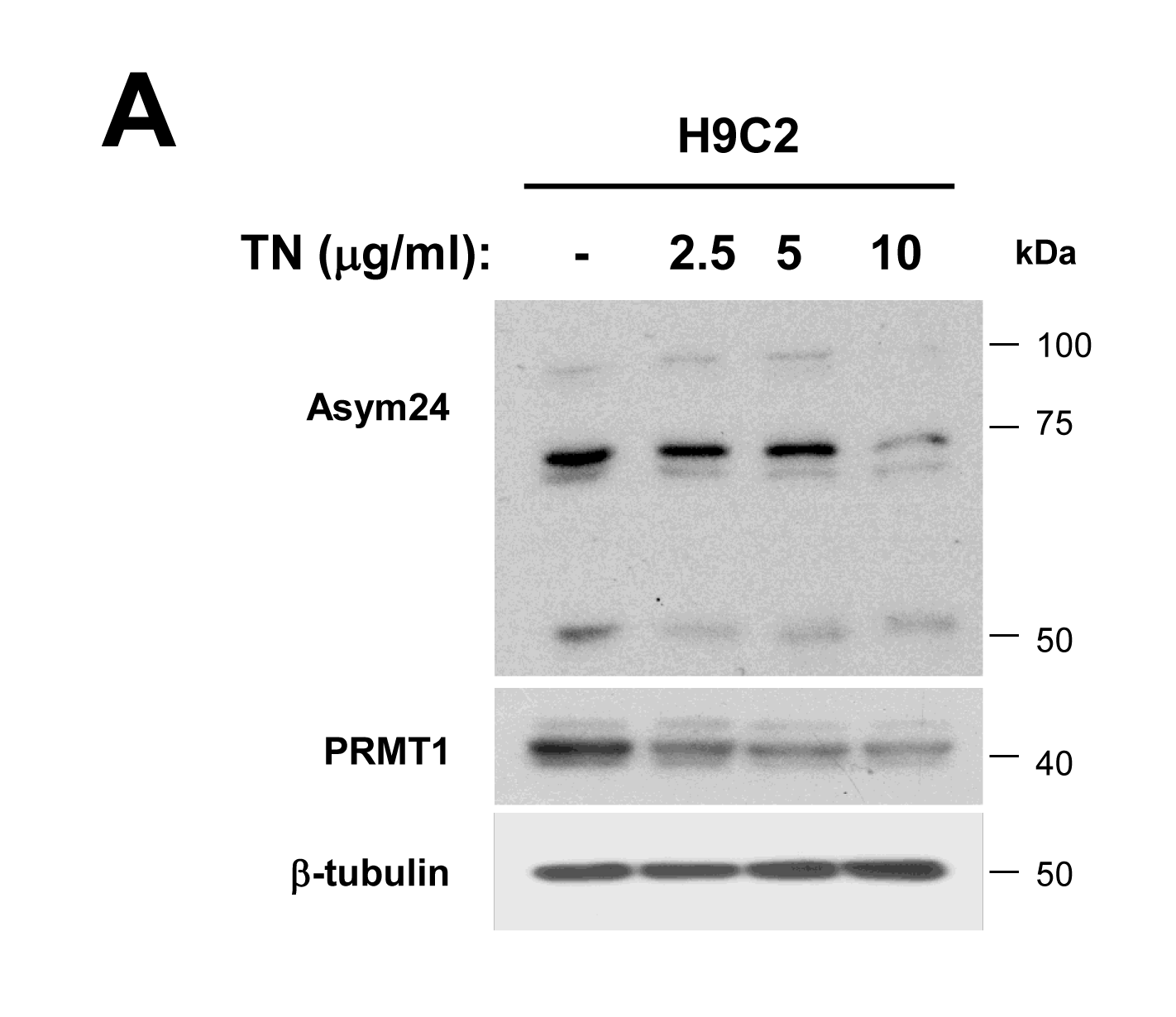

Supplement: Supplementary file 3 — Figure 2s [file 41419_2019_2147_MOESM3_ESM.tif]

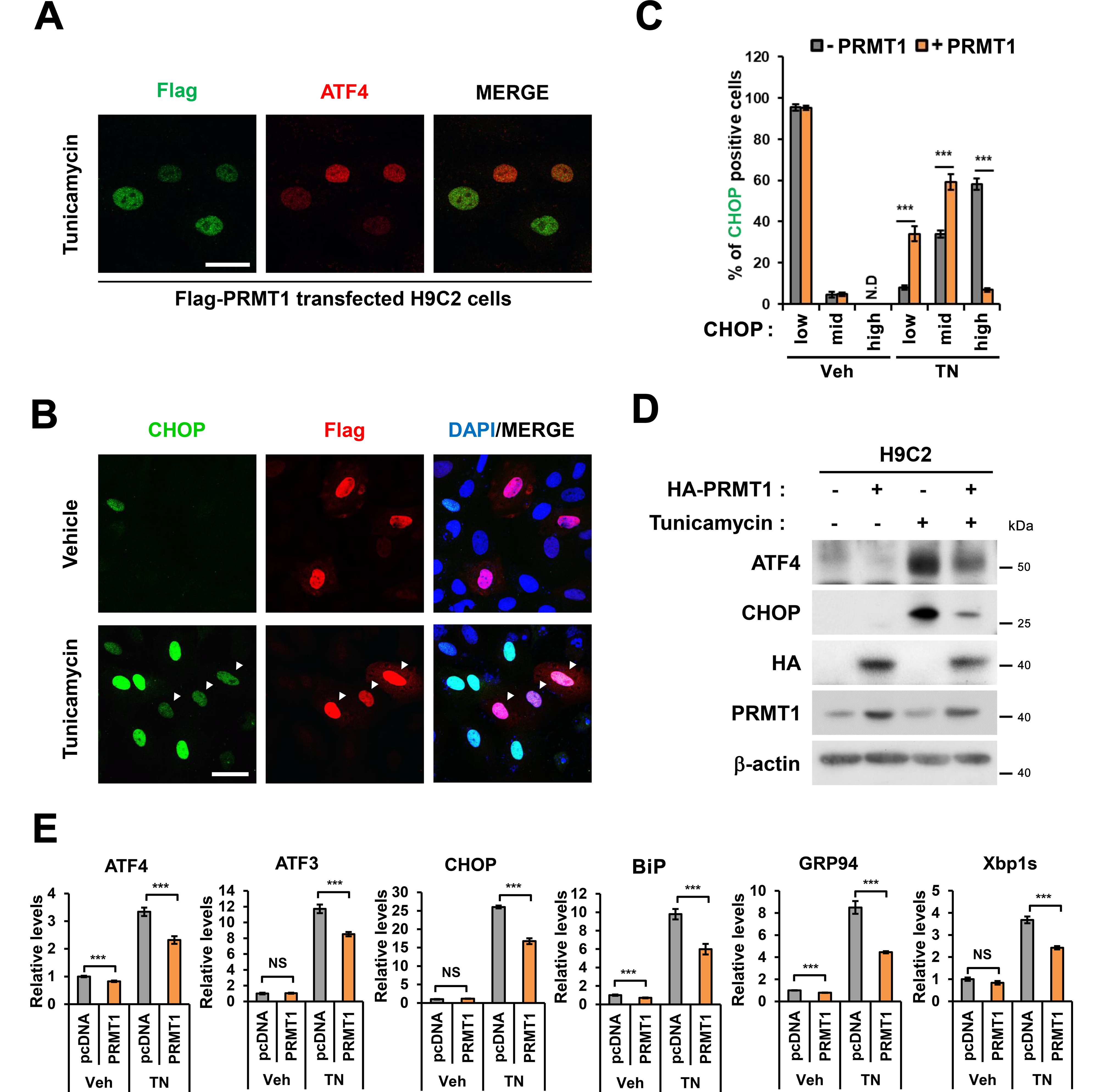

Supplement: Supplementary file 4 — Figure 3s [file 41419_2019_2147_MOESM4_ESM.tif]

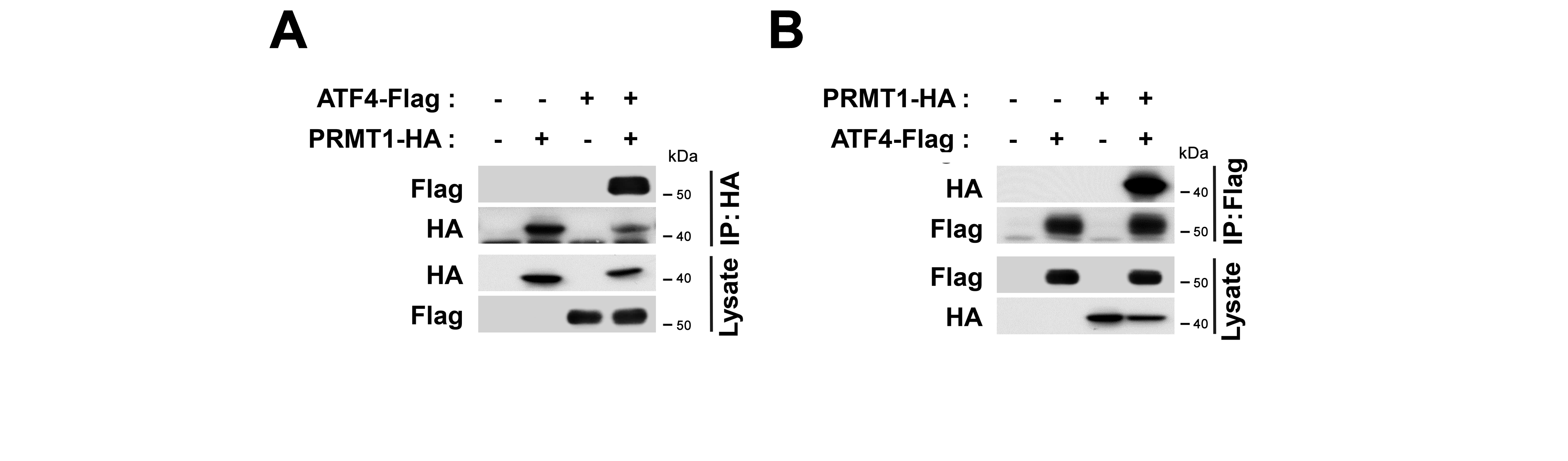

Supplement: Supplementary file 5 — Figure 4s [file 41419_2019_2147_MOESM5_ESM.tif]

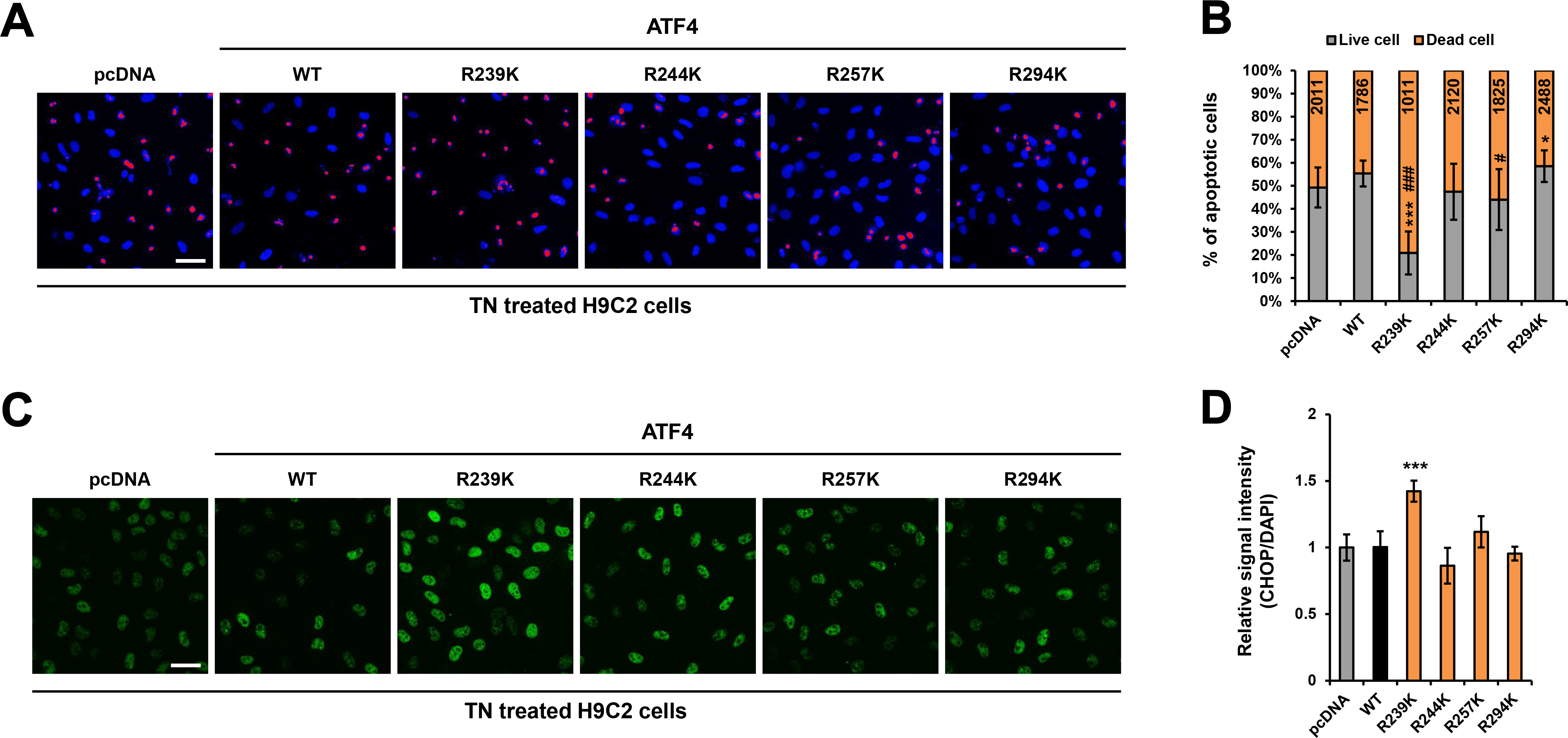

Supplement: Supplementary file 6 — Figure 5s [file 41419_2019_2147_MOESM6_ESM.tif]

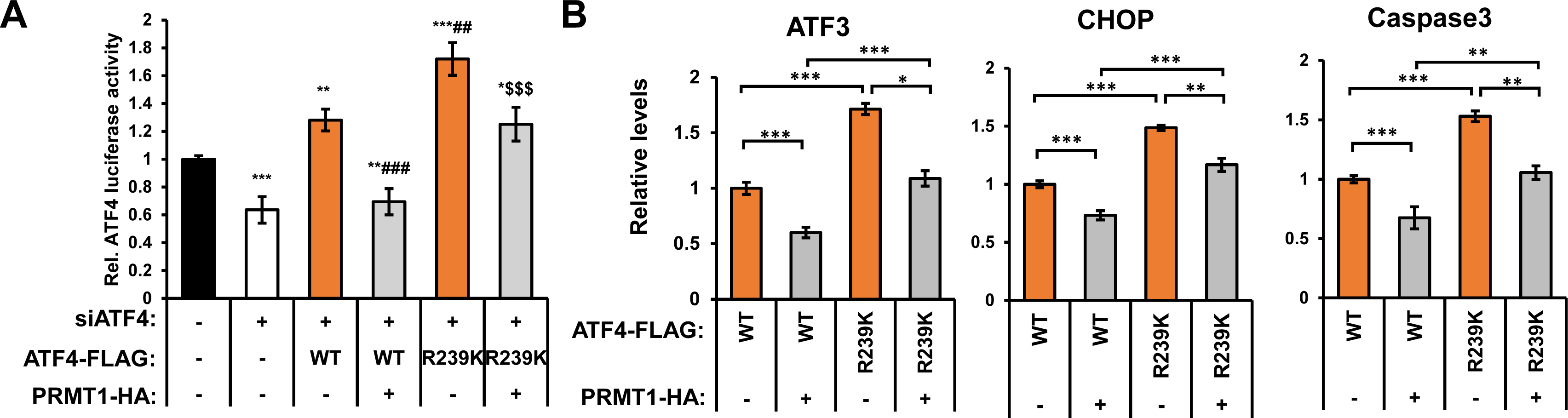

Supplement: Supplementary file 7 — Figure 6s [file 41419_2019_2147_MOESM7_ESM.tif]
