# Supplementary material for: PRMT1 suppresses ATF4-mediated endoplasmic reticulum response in cardiomyocytes
Source: Cell Death Dis. 2019 Dec 2;10(12):903. doi: 10.1038/s41419-019-2147-3 (PMC6885520; doi:10.1038/s41419-019-2147-3)
Supplement: Supplementary file 8 — Supplementary Tables [file 41419_2019_2147_MOESM8_ESM.docx]

**Supplementary Table 1s. The primary antibodies used in this study.**

| Antigen | Host | Cat. No. | Manufacturer |
| --- | --- | --- | --- |
| Asym24 | R | 13522S | Cell signaling technology |
| ATF4 | R | 11815S | Cell signaling technology |
| ATF6 | M | ab122897 | Abcam |
| β-actin | R | 4970L | Cell signaling technology |
| BiP | R | 3177S | Cell signaling technology |
| β-tubulin | M | 32-2600 | Invitrogen |
| c-Caspase3 | R | 9664S | Cell signaling technology |
| CHOP | M | 2895S | Cell signaling technology |
| eIF2α | R | 5324S | Cell signaling technology |
| Flag | M | F1804 | Sigma-Aldrich |
| HA-HRP | R | PA1-29751 | Thermo Fisher Scientific |
| HSP90 | R | Sc-7947 | Snta Cruz |
| p-eIF2α | R | 3398S | Cell signaling technology |
| p-γH2AX | R | 9718S | Cell signaling technology |
| PRMT1 | M | 07-404 | Merck Millipore |
| Ub-K48 | R | 15-1307 | Merck Millipore |
| GAPDH | R | LF-PA0018 | AbFrontier |
| p-CaMKII (T286) | R | Ab32678 | Abcam |
| t-CaMKII | R | 3362S | Cell signaling technology |

**Supplementary Table 2s.** **The primer sequence for human ATF4 mutagenesis.**

| Name | 5' to 3' |
| --- | --- |
| ATF4 R239K-F | GCA CAG CCC CTC TAC CAA GGG CTC TCC |
| ATF4 R239K-R | GGA GAG CCC TTG GTA GAG GGG CTG TGC |
| ATF4 R244K-F | CCA GGG GCT CTC CAA ATA AGA GCC TCC CA |
| ATF4 R244K-R | TGG GAG GCT CTT ATT TGG AGA GCC CCT GG |
| ATF4 R257K-F | TGT TCT CTG TGG GTC TGC CAA GCC CAA ACC TTA CGA TCC TC |
| ATF4 R257K-R | GAG GAT CGT AAG GTT TGG GCT TGG CAG ACC CAC AGA GAA CA |
| ATF4 R294K-F | AAG ACA GCA GCC ACT AAG TAC CGC CAG AAG AAG |
| ATF4 R294K-R | CTT CTT CTG GCG GTA CTT AGT GGC TGC TGT CTT |

**Supplementary Table 3s.** **The primer sequence for qRT-PCR analysis.**

| Gene symbol |  | 5' to 3' |
| --- | --- | --- |
| ACTB | Forward | CATGTACGTTGCTATCCAGGC |
|  | Reverse | CTCCTTAATGTCACGCACGAT |
| NOXA | Forward | ACCAAGCCGGATTTGCGATT |
|  | Reverse | ACTTGCACTTGTTCCTCGTGG |
| Caspase3 | Forward | TGA CTG GAA AGC CGA AAC TC |
|  | Reverse | AGC CTC CAC CGG TAT CTT CT |
| ATF3 | Forward | ATG TCC TCT GCG CTG GAG T |
|  | Reverse | ACA CTT GGC AGC AGC AAT TT |
| ATF4 (R) | Forward | AAA CCT CAT GGG TTC TCC AG |
|  | Reverse | TCT CCA ACA TCC AAC TGT CC |
| ATF4 (M) | Forward | TGG GGC CTT TAG GAC GAT CT |
|  | Reverse | GCC CTA AAC CCG CCC TTT AT |
| CHOP | Forward | TAT CTC ATC CCC AGG AAA CG |
|  | Reverse | CAG GGT CAA GAG TAG TGA AGG TTT |
| XBP1s | Forward | TGC TGA GTC CGC AGC AGG TG |
|  | Reverse | ACA GGG TCC AAC TTG TCC AG |
| GADD34 | Forward | GGA CCC TGA GAT TCC TCT GA |
|  | Reverse | GCC CAG ACA GCA AGG AAA T |
| GRP78 | Forward | TGC AGC AGG ACA TCA AGT TC |
|  | Reverse | TTT CTT CTG GGG CAA ATG TC |
| GRP94 | Forward | TGC TTC TGA TGC TTT AGA CAA GA |
|  | Reverse | CTG TGA CAT GCA GCA GGT TT |
